# Non-placement versus placement of a drainage tube around the cervical anastomosis in McKeown esophagectomy: study protocol for a randomized controlled trial

**DOI:** 10.1186/s13063-019-3750-4

**Published:** 2019-12-23

**Authors:** Taro Oshikiri, Gosuke Takiguchi, Susumu Miura, Nobuhisa Takase, Hiroshi Hasegawa, Masashi Yamamoto, Shingo Kanaji, Kimihiro Yamashita, Yoshiko Matsuda, Takeru Matsuda, Tetsu Nakamura, Satoshi Suzuki, Yoshihiro Kakeji

**Affiliations:** 1 Division of Gastrointestinal Surgery, Department of Surgery, Graduate School of Medicine, Kobe University, 7-5-2, Kusunoki-cho, Chuo-ku, Kobe, Hyogo 650-0017 Japan; 2 Division of Minimally Invasive Surgery, Department of Surgery, Graduate School of Medicine, Kobe University, 7-5-2, Kusunoki-cho, Chuo-ku, Kobe, Hyogo 650-0017 Japan; 3 Department of Social Community Medicine and Health Science, Division of Community Medicine and Medical Network, Graduate School of Medicine, Kobe University, 7-5-2, Kusunoki-cho, Chuo-ku, Kobe, Hyogo 650-0017 Japan

**Keywords:** McKeown esophagectomy, Cervical drainage tube, Anastomotic leakage

## Abstract

**Background:**

Esophagectomy with extended lymphadenectomy remains the mainstay of treatment for localized esophageal cancer. Currently, transthoracic and abdominal esophagectomy with cervical anastomosis (McKeown esophagectomy) is a frequently used technique in Japan. However, cervical anastomosis is still an invasive procedure with a high incidence of anastomotic leakage. The use of a drainage tube to treat anastomotic leakage is effective, but the routine placement of a closed suction drain around the anastomosis at the end of the operation remains controversial. The objective of this study is to evaluate the postoperative anastomotic leakage rate, duration to oral intake, hospital stay, and analgesic use with nonplacement of a cervical drainage tube as an alternative to placement of a cervical drainage tube.

**Methods:**

This is an investigator-initiated, investigator-driven, open-label, randomized controlled parallel-group, noninferiority trial. All adult patients (aged ≥20 and ≤85 years) with histologically proven, surgically resectable (cT1–3 N0–3 M0) squamous cell carcinoma, adenosquamous cell carcinoma, or basaloid squamous cell carcinoma of the intrathoracic esophagus, and European Clinical Oncology Group performance status 0, 1, or 2 are assessed for eligibility. Patients (*n* = 110) with resectable esophageal cancer who provide informed consent in the outpatient clinic are randomized to either nonplacement of a cervical drainage tube (*n* = 55) or placement of a cervical drainage tube (*n* = 55).

The primary outcome is the percentage of Clavien–Dindo grade 2 or higher anastomotic leakage.

**Discussion:**

This is the first randomized controlled trial comparing nonplacement versus placement of a cervical drainage tube during McKeown esophagectomy with regards to the usefulness of a drain for anastomotic leakage. If our hypothesis is correct, nonplacement of a cervical drainage tube will be recommended because it is associated with a similar anastomotic leakage rate but less pain than placement of a cervical drainage tube.

**Trial registration:**

UMIN-CTR, 000031244. Registered on 1 May 2018.

## Background

Torek described the world’s first case of transthoracic esophagectomy in 1913 [1]. Esophagectomy was performed via the left thoracic cavity without anastomosis. The meal passed from the stoma of the proximal esophagus through an external tube to the gastrostomy. Esophagectomy with extended lymphadenectomy has remained the mainstay of treatment for localized esophageal cancer [2–4]. Esophagectomy currently involves two surgical anastomoses: the McKeown approach (cervical anastomosis) [5] and the Ivor Lewis approach (intrathoracic anastomosis). Currently, transthoracic and abdominal esophagectomy with cervical anastomosis (McKeown esophagectomy) is the more commonly used technique in Japan. However, cervical anastomosis is still an invasive approach with a high incidence of anastomotic leakage [6–8]. The use of a drainage tube as treatment for anastomotic leakage is effective [9], but the efficacy of routinely placing a closed suction drain around the anastomosis at the end of the operation remains controversial. To date, there have been few prospective randomized controlled trials comparing nonplacement versus placement of a cervical drainage tube during McKeown esophagectomy. Choi et al. reported a randomized trial to evaluate the role of a drainage tube for the esophageal cervical anastomosis in 40 patients. They concluded that routine use of a neck drain for esophageal anastomosis in the neck is not necessary as there were no anastomotic leaks, seromas or hematomas in either group [10]. However, the number of patients in their study was too small to evaluate the usefulness of a drain for anastomotic leakage. Additionally, a cervical drainage tube might lead to respiratory complications including pneumothorax if it were placed from the neck into the thorax [11].

We present the protocol for the randomized controlled trial comparing these two surgical treatments.

## Methods/design

### Study aim

This is a randomized controlled, parallel-group, noninferiority trial of nonplacement versus placement of a cervical drainage tube in patients who undergo McKeown esophagectomy for indication.

### Objectives

Patients with resectable esophageal cancer are randomized in the outpatient clinic to either the nonplacement or placement group. The objective is to evaluate the safety and efficacy of nonplacement of a cervical drainage tube as an alternative to predictive placement of a cervical drainage tube as treatment for anastomosis leakage in McKeown esophagectomy. We hypothesize that nonplacement of a cervical drainage tube leads to a noninferior postoperative anastomotic leakage rate, duration to oral intake, hospital stay, and less analgesic use compared with placement of a cervical drainage tube, which is the current standard of care.

### Study design

This is an investigator-initiated, investigator-driven, open-label, randomized controlled, parallel-group, noninferiority trial comparing nonplacement versus placement of a cervical drainage tube during esophagectomy with cervical anastomosis.

This study will be conducted in compliance with the Declaration of Helsinki [12] and the Kobe University Conflicts of Interest Management Guidelines. Written informed consent will be obtained from all participating patients. The principal investigator will appoint responsible monitors for this study. The appointed monitors must have received training on the Ethical Guidelines for Medical and Health Research involving Human Subjects and other regulatory requirements and be thoroughly familiar with the contents of the study protocol, informed consent form, and written monitoring procedures.

### Study population

The diagnosis of esophageal cancer was based on the seventh edition of the Union for International Cancer Control tumor node metastasis cancer staging system [13]. All Japanese adult patients (aged ≥20 and ≤85 years) with histologically proven, surgically resectable (cT1–3 N0–3 M0) squamous cell carcinoma, adenosquamous cell carcinoma, or basaloid squamous cell carcinoma of the intrathoracic esophagus will be assessed for eligibility. Patients should have European Clinical Oncology Group performance status of 0, 1, or 2.

The inclusion criteria are:

- Histologically proven squamous cell carcinoma, adenosquamous cell carcinoma, or basaloid squamous cell carcinoma of the intrathoracic esophagus
- Surgically resectable disease (T1–3 N0–3 M0)
- Age ≥20 and ≤85 years
- European Clinical Oncology Group performance status of 0, 1, or 2
- Written informed consent

The exclusion criteria are:

- Carcinoma of the cervical esophagus
- Carcinoma of the gastroesophageal junction with the majority of the tumor in the gastric cardia
- Severe systemic infection
- Pregnancy
- Mental illness
- Systemic steroid or immunosuppressive therapy

### Study protocol

Patients are informed about the trial by one of our surgeons (TO) in the Surgical Oncology outpatient clinic. After receiving information about the trial, all patients have 2 weeks to consider whether to participate. After 2 weeks, the coordinating researcher (YK) contacts the patient to see if they would like to make an appointment to provide informed consent. After obtaining informed consent, randomization is performed using a computerized random number generator by a researcher (KY) on the eve of the operation. Concealment of allocation is maintained by using sealed opaque envelopes. There is no blinding for the patient, surgeon, or coordinating researcher after the operation because this is difficult in daily practice. After the operation, patients are informed about the allocated treatment. This study is completely funded by the Division of Gastrointestinal Surgery of Kobe University. The patient’s clinical status is assessed, and preoperative testing is performed.

Next, patients undergo the randomized intervention with either nonplacement or placement of a cervical drainage tube. The study began on 26 July 2018. Each patient undergoes follow-up at 2 weeks after surgery to assess primary and secondary outcomes (Table 1).

**Table 1:**
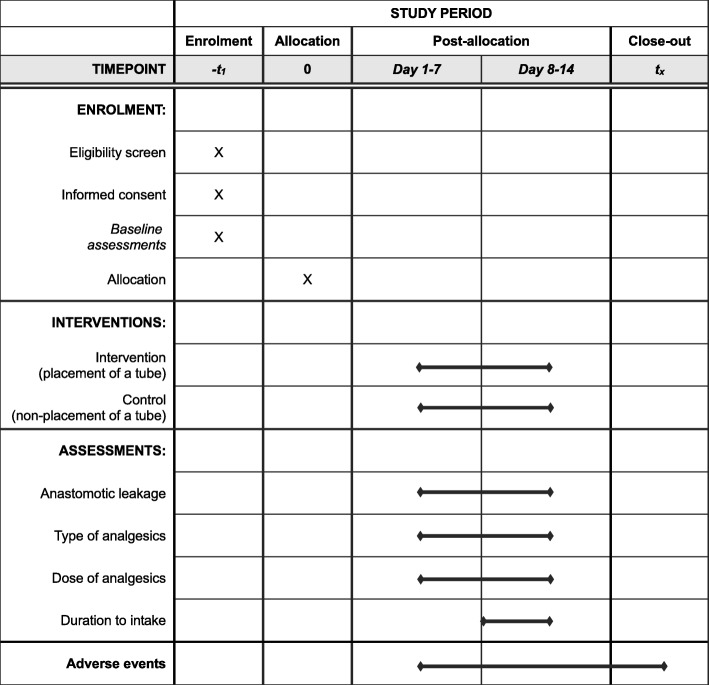
Perioperative outcome parameters and schedule of study visits and follow-up

### Criteria for discontinuation

When a participant’s continuation in the study is judged to be impossible (see below for possible reasons), the principal investigator or subinvestigator will withdraw the participant from the study and specify the date and time of the discontinuation or dropout, the reason for discontinuation or dropout, and the clinical course in the medical record and case report form (CRF). In addition, at the time of the discontinuation/dropout, the principal investigator or subinvestigator will perform necessary tests and assess the efficacy and safety.

Reasons for discontinuation or dropout include:

- When the participant requests to withdraw from the study or withdraws consent
- When the participant is found not to meet the eligibility criteria after enrollment
- When a concurrent disease worsens and further continuation with the study is difficult
- When an adverse event occurs and further continuation with the study is difficult
- When discontinuation from the study is appropriate for other reasons in the opinion of the principal investigator or subinvestigator

### Handling of adverse events at onset

#### Definition of adverse events

An adverse event is defined as any unfavorable or unintended sign (including an abnormal laboratory change), symptom, or disease occurring after treatment has been allocated, whether or not related to the study treatment.

#### Action taken for the participant following onset of an adverse event

When an adverse event is noted, the principal investigator or subinvestigator will immediately perform appropriate treatment and will record the adverse event in the medical record and CRF without discrepancies. In addition, if the adverse event requires treatment, this should be explained to the participant.

#### Reportable adverse events

All adverse events occurring no later than 14 days after the allocated treatment must be reported, irrespective of the causal relationship to the study treatment, and followed up until resolution or up to 4 weeks after the end (or discontinuation) of the study period. All adverse events assessed to be related to the study drug must be reported through to the end of the study period.

#### Procedures for reporting an adverse event following onset

The principal investigator or subinvestigator will record all adverse events occurring during the abovementioned period in the medical record and CRF without discrepancies. The adverse events should be followed up as far as possible until recovery of the participant to the baseline status or no further follow-up is judged to be required.

#### Information to be recorded for assessment of adverse events

The following information is required to be recorded for assessment of adverse events:

1. Adverse event term
2. Date of onset
3. Date of outcome
4. Outcome: resolved/resolving/resolved with sequelae/not resolved/death/unknown
5. Action taken (for the study drug): no change/discontinued/suspended/dose reduction/dose increase/not applicable
6. Other action taken: none/pharmacotherapy/other
7. Seriousness: nonserious/serious
8. Severity: mild/moderate/severe
9. Causal relationship to the study drug: related/not related

### Handling of serious adverse events following onset

#### Definition of serious adverse events

A serious adverse event is defined as any event that:

1. Results in death
2. Is life-threatening
3. Results in disability (i.e., dysfunction that interferes with activities in daily living)
4. Results in potential disability
5. Requires hospitalization or prolongation of existing hospitalization for treatment
6. Is serious on the lines of (1) to (5) above
  or
7. Is a congenital disease or anomaly in the offspring

#### Reportable serious adverse events

Reporting is required for all serious adverse events occurring during the study period, as well as serious adverse events occurring no later than 14 days after the end (or discontinuation) of the study and suspected to be related to the study drug.

#### Procedures for reporting serious adverse events

If an adverse event has occurred and is considered to be serious by the principal investigator, the adverse event data should be handled according to the following procedures:

1. Reporting from the principal investigator to the head of the research institution and the study representative. The principal investigator will report the adverse event data to the head of the research institution and the study representative as soon as possible, irrespective of the causal relationship to the study drug.
2. Action taken upon receipt of additional information. When any additional information is obtained regarding the adverse event, the principal investigator of the research institution involving the adverse event will report the additional information to the head of the research institution as soon as possible.

### Surgical procedures

#### Thoracic procedure

All patients undergo thoracoscopic esophagectomy in the prone position with radical esophagectomy and total mediastinal lymphadenectomy. To permit easy retraction of the trachea, a single-lumen tracheal tube is inserted into the trachea and a blocker is inserted into the right bronchus for one-lung anesthesia before the procedure. The patient is initially placed in the prone position. Six 5-mm or 12-mm ports are inserted into the third intercostal space (ICS) posterior to the midaxillary line, the fifth and seventh ICS on the posterior axillary line, the sixth and eighth ICS on the midaxillary line, and the ninth ICS on the scapular angle line for fine lymphadenectomy, dividing the esophagus, and dividing the azygos arch. The chest cavity is inflated via the ports with carbon dioxide to an insufflation pressure of 6–8 mmHg. The endoscope is usually inserted through the port in the ninth ICS.

#### Abdominal and cervical procedures

The abdominal procedure is performed completely laparoscopically. Gastric mobilization, abdominal lymphadenectomy around the left gastric pedicle and the celiac axis, and excision of the entire isolated thoracic esophageal specimen and dissected lymph nodes through the esophageal hiatus are initially performed. Next, a gastric conduit of 3–4 cm in width is created outside of the wound and raised via the posterior mediastinum. Subsequently, the esophagogastric anastomosis is made in the neck. For three-field lymph node dissection, the cervical nodes are removed through a collar incision.

#### Placement of cervical drainage tube around the cervical anastomosis

Patients assigned to the placement group undergo placement of a 15-Fr silicone drain through the skin near the left cervical incision. The tip of the drain is placed near the anastomosis. If there are no signs of leakage, the drain is removed on postoperative day 7. Anastomotic leakage will be diagnosed by flare of the neck skin, inflammatory response in a blood examination, nature of the drainage fluid, and findings of computed tomography [14].

#### Study device

A 15-Fr BLAKE Silicone Drain (Ethicon, NJ, USA) is placed around the cervical anastomosis in the placement group. The BLAKE Silicone Drain is made of silicone. The entire drain is flexible and has channels along the sides, instead of holes, to facilitate drainage. It is always used with a continuous suction device (J-VAC Suction Reservoir; Ethicon), which creates a closed drainage system. Drainage is very efficient; a larger area is in contact with the tissue as compared with perforated drains, and the fluid is efficiently removed by capillary pressure.

#### Expected adverse events for medical devices

Hemorrhage, pain, and ascending infection may occur in some cases.

#### Safety and adverse event monitoring

Gastrointestinal surgeons and statisticians are responsible for overseeing the progress and safety of the study, including monitoring adverse events, morbidity, and withdrawals from the study. All adverse events are evaluated for severity. Any serious adverse events such as death, disability, and prolonged hospitalization will be recorded on the CRF and reported to the Biological and Medical Ethics Committee of Kobe University Hospital within 24 h. If the number of serious adverse events related to treatment is higher than in our own database or as reported by other authors, patient enrollment will be terminated immediately, and the Medical Ethics Committee will reassess the safety of the trial.

### Outcome measurements

#### Primary outcome

The primary outcome of this study is the percentage of anastomotic leakage events (grade 2 or higher) based on the Clavien–Dindo classification of surgical complications [15].

#### Secondary outcomes

The duration from surgery to oral intake, hospital stay, and type and dose of analgesics used during hospitalization will be assessed.

#### Interim analysis

No interim analysis is planned in this study.

#### Independent Data Monitoring Committee

An Independent Data Monitoring Committee (IDMC) will be established for this study. The IDMC will be formed as an organization independent from the sponsor/investigator, and will consist of at least two members who are specialists and independent of this study. The IDMC will conduct safety monitoring, including comparison of the incidence of adverse events between the test treatment and control and detailed investigation of serious adverse events as necessary, in order to secure the safety of the patients. Based on the safety monitoring results, the IDMC may advise a change to the study design (e.g., change of the inclusion criteria) to reduce the risk of certain adverse events or discuss appropriateness of continuation of the study.

The IDMC will discuss the appropriateness of continuation of the study based on the regular monitoring. On the basis of this discussion, the IDMC will advise the sponsor/investigator on the appropriateness of continuing the study and the appropriateness of publishing the study results.

### Sample size calculation

**Fig. 1:**
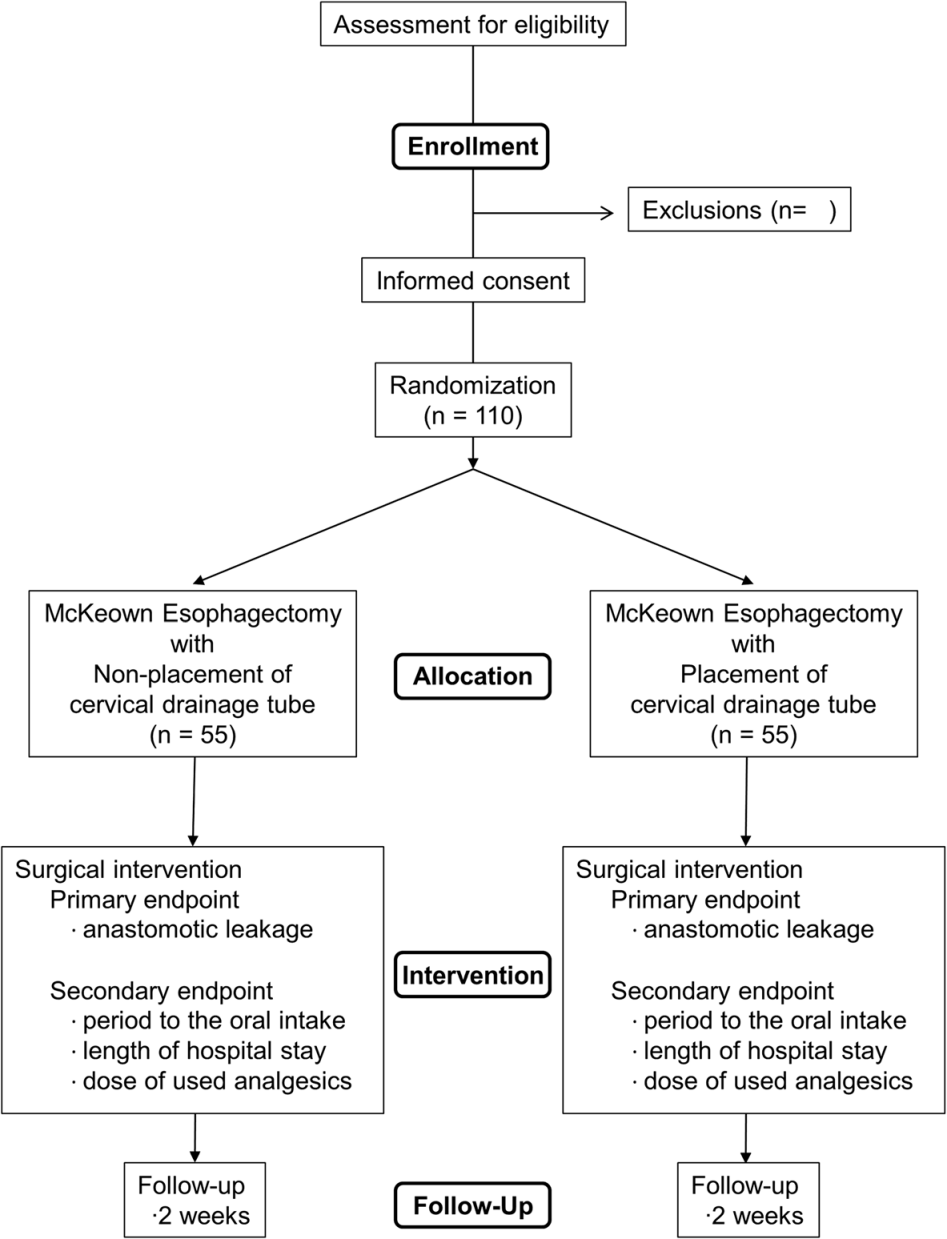
Flowchart for the cervical drainage tube trial

Based on data from our own case series, the rate of Clavien–Dindo grade 2 or higher anastomosis leakage is 5%. Noninferiority is defined as a significant difference in the primary outcome between the two arms of less than 12%. Based on a one-sided type 1 error rate of 2.5% and power of 80%, the sample size needed to exclude a noninferiority margin of 12% for the difference in the proportion of participants reaching the primary outcome is 104 patients. An estimated compensation of 5% for withdrawals was added, resulting in a total of 110 patients, with 55 in each arm. Figure 1 summarizes the final study design.

### Statistical analysis

All prospective data will be statistically analyzed using JMP® 11 (SAS Institute Inc., Cary, NC, USA). To evaluate the significance of differences between the two groups, Chi-squared and Fisher’s exact tests will be used as appropriate for categorical variables, and the nonparametric Mann–Whitney *U* test will be used for continuous variables. The significance level will be set at 5%.

### Protocol amendments

If important protocol modifications (e.g., changes to eligibility criteria, outcomes, analyses) are required, we will communicate these to relevant parties (e.g., investigators, Research Ethics Committees/Institutional Review Boards, trial participants, trial registries, journals, regulators) by email, telephone, and letter.

### Consideration of human rights (privacy protection)

For de-identification of participant data, the principal investigator or subinvestigator will remove personally identifiable information (e.g., participant’s name, initials, address, telephone number, medical record number) from the data management. A Subject Identification Code will be used for the enrollment of the participant and completion of the CRF, and so on.

### Final analysis

The final analysis will be performed after data from the participants have been obtained and locked after the end of the follow-up period. The responsible biostatistician will prepare the statistical analysis report and submit it to the principal investigator.

### Ownership and publication of study results

The study results will be presented at a medical society meeting and then sent for publication in a medical journal in English. As necessary, the results will also be presented at Japanese medical society meetings.

## Discussion

Randomized clinical trials have demonstrated that prophylactic drains do not decrease the incidence of postoperative complications in elective hepatectomy, colectomy, and cholecystectomy [16–19]. In pancreatic head resection, drain removal on postoperative day 4 was shown to be independently associated with a lower incidence of complications, including intra-abdominal infections [20]. Ascending infections along the drain may increase the incidence of pancreatic fistula in patients with long-term drain placement. On the other hand, whether to place a drain near the cervical anastomosis after McKeown esophagectomy remains controversial, even though drains usually are inserted near the cervical anastomosis at most institutions. In 1998, Choi et al. [10] reported a randomized trial to evaluate the role of closed suction drainage for esophageal anastomosis in the neck. In that randomized controlled study of 40 patients with esophageal carcinoma who underwent esophagectomy with an esophageal anastomosis in the neck, half had a neck drain inserted at the end of the operation. The median duration of drainage was 46 h (range 36–88 h). The median amount of drainage was 63 ml (range 15–210 ml). No hematoma or seroma formation occurred in either group. Anastomotic leakage did not occur in any patients. Consequently, the benefits of closed suction neck drainage could not be demonstrated. They concluded that routine use of a neck drain for esophageal anastomosis in the neck is not necessary [10]. However, there were some limitations in that study. First, the number of patients was too small to evaluate the usefulness of a drain for anastomotic leakage as we described in the section on sample size calculation. Second, their cohort might not have been representative of patients who undergo esophagectomy in general because none of the patients had anastomotic leakage; in general, the anastomotic leakage rate in published studies ranges from 1.5% to 21% [21–25]. In the current study, we hypothesize that nonplacement of a cervical drainage tube will lead to a noninferior postoperative anastomotic leakage rate. As a result, we can omit routine placement of a cervical drainage tube which should contribute to reduce cervical pain. Conversely, if a cervical drainage tube does indeed drain the digestive juices when anastomotic leakage occurs, the Clavien–Dindo grade may improve from grade 3 or higher to grade 2. If so, we can confirm the importance of placing a cervical drainage tube around the anastomosis during McKeown esophagectomy. This randomized trial can provide further evidence to support the use or omission of cervical drainage tube placement in patients who undergo McKeown esophagectomy for esophageal cancer.

## Conclusions

This is the first randomized controlled trial comparing nonplacement versus placement of a cervical drainage tube during McKeown esophagectomy with respect to the usefulness of a drain if anastomotic leakage occurs. If our hypothesis is correct, nonplacement of a cervical drainage tube will be recommended since the anastomotic leakage rate would be similar but there would be less pain compared with the current standard care using a cervical drainage tube.

## Trial status

This is protocol version 2.1, 30 March 2018. Recruitment began on 30 March 2018, and is expected to be completed on or around 31 October 2020.

## Supplementary information

**Additional file 1.** SPIRIT 2013 Checklist: Recommended items to address in a clinical trial protocol and related documents.

## Data Availability

The datasets obtained and/or analyzed during the current study will be available from the corresponding author on reasonable request.

## Abbreviations

CRF: Case report form
ICS: Intercostal space
IDMC: Independent Data Monitoring Committee
